# Kinetics of drug selection systems in mouse embryonic stem cells

**DOI:** 10.1186/1472-6750-13-64

**Published:** 2013-08-07

**Authors:** Yuhki Nakatake, Setsuko Fujii, Shinji Masui, Toshimi Sugimoto, Satomi Torikai-Nishikawa, Kenjiro Adachi, Hitoshi Niwa

**Affiliations:** 1 Laboratory for Pluripotent Stem Cell Studies, RIKEN Center for Developmental Biology, 2-2-3 Minatojima-minamimachi, Chuo-ku, Kobe 650-0047, Japan; 2 Department of Systems Medicine, Sakaguchi Laboratory, Keio University School of Medicine, 35 Shinanomachi, Shinjyuku-ku, Tokyo 160-8582, Japan; 3 Department of Reprogramming Science, Center for iPS cell Research and Application, Kyoto University, 53 Syogoin-Kawaharacho, Sakyo-ku, Kyoto 606-8507, Japan; 4 Laboratory for Development and Regenerative Medicine, Kobe University Graduate School of Medicine, 7-5-1 Kusunokicho, Chuo-ku, Kobe 650-0017, Japan; 5 JST, CREST, Sanbancho, Chiyoda-ku, Tokyo 102-0075, Japan

**Keywords:** Transgene, Expression, Marker, Gene targeting, Vector

## Abstract

**Background:**

Stable expression of transgenes is an important technique to analyze gene function. Various drug resistance genes, such as *neo, pac, hph, zeo, bsd,* and *hisD*, have been equally used as selection markers to isolate a transfectant without considering their dose-dependent characters.

**Results:**

We quantitatively measured the variation of transgene expression levels in mouse embryonic stem (mES) cells, using a series of bi-cistronic expression vectors that contain *Egfp* expression cassette linked to each drug resistant gene via *IRES* with titration of the selective drugs, and found that the transgene expression levels achieved in each system with this vector design are in order, in which *pac* and *zeo* show sharp selection of transfectants with homogenously high expression levels. We also showed the importance of the choice of the drug selection system in gene-trap or gene targeting according to this order.

**Conclusions:**

The results of the present study clearly demonstrated that an appropriate choice of the drug resistance gene(s) is critical for a proper design of the experimental strategy.

## Background

The introduction of exogenous transgene cassettes into culture cells to direct their expressions is an important strategy in molecular biology to analyze the functions of the genes. However, a simple introduction of the DNA fragment into cells by either electroporation or lipofection results in its stable integration into the genome of the host cells only at a low frequency. Therefore, it is always required to select the cells carrying the integrated copies of the transgenes by using dominant selection markers. The combinations of the antibiotics that kill the mammalian cells and the genes that establish the resistance against them have been preferentially applied for this purpose: such as *neomycin phosphotransferase II* from transposon *Tn5* (designated as *neo* in this paper) against the neomycin derivative G418, *puromycin N-acetyltransferase* from *Streptomyces alboniger* (*pac*) against puromycin, *hygromycin B phosphotransferase* from *Escherichia coli* (*hph*) aginst hygromycin B*, Streptoalloteichus hindustanus ble* (*Sh ble*: designated as *zeo* in this paper) against the bleomycin derivative zeocin*, blasticidin S deaminase* from *Aspergillus terreus* (*bsd*) against blasticidin S, and *histidinol dehydrogenase* from *Salmonella typhimurium* (*hisD*) against histidinol [1-6]. These drugs and the resistance genes have equally been regarded as dominant selection markers that reflect the introduction of the transgenes into mammalian cells. Transfection of drug resistance genes together with transgenes, each in separate expression cassette, to obtain stable transfectants has been a commonly used method. However, in this strategy, the drug resistance does not always appropriately reflect the expression level of the transgene because generally the stable expression levels of exogenous expression cassettes are highly sensitive to thier sites of integration, as a result of the local chromatin environment when the transgenes are randomly integrated into the host genome [7], which affect the expression levels of the drug resistance gene cassette and the transgene cassette separately.

The bi-cistronic expression of the transgene and the drug resistance gene using an internal ribosome entry site (*IRES*) is able to confer a tight correlation between the transgene expression and the drug resistance because the *IRES*-mediated cap-independent translation ensures parallel expressions of the transgene and the drug resistance gene [8]. This vector design is particularly important to drive transgene expressions in mouse embryonic stem (mES) cells since the silencing effect to the stably integrated transgene cassette is problematic in these cells [9]. In this vector design, the expression levels of the transgenes depend on the threshold expression levels of the drug resistance genes that confer the survival of the transfectants in the presence of the drugs. The expression levels of some transgenes can also possess unique thresholds based on their effect on cell viability. The combination of the effect of the transgene with the range of selection generated by the antibiotic resistance marker can produce a narrow expression range that can mimic the physiological function range of expression. Therefore, a proper choice of drug resistance genes is important to achieve the optimal range of transgene expression levels.

Here we demonstrated the parallel comparison of the kinetics among each drug selection system determined by the expression levels from the *IRES*-based expression vectors. We found obvious differences in their kinetics and the impact on various experimental situations.

## Results

### Kinetics of the drug-selection systems in the *IRES*-based expression vectors

**Figure 1:**
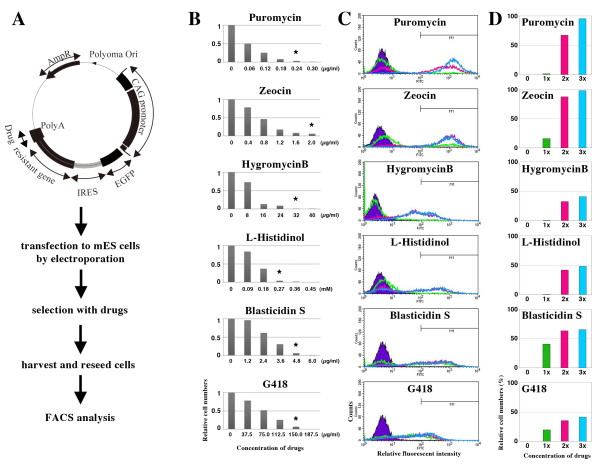
**Expression levels of the transgenes from the stably integrated bi-cistronic transgene cassettes with various drug selection systems. (A)** Design of the bi-cistronic expression vectors containing the drug resistance genes. The drug resistance genes for G418 (*neo*), puromycin (*pac*), hygromycin B (*hph*)*,* zeocin (*zeo*)*,* blasticidin S (*bsd*), and histidinol (*hisD*) were fused to *EMCV-IRES* and placed downstream of *Egfp* under the control of the *CAG* expression unit [10]. **(B)** Determination of the minimal concentrations of the drugs. The D3 mES were seeded at low cell density (1000 cells per 90 mm dish) with various concentrations of drugs in triplicate, and the cell numbers were counted. The relative number of the cells to that obtained without the drug, which was set at 1.0, are shown. Asterisks show the doses of 1× of the selection in FACS analyses, by which more than 93% of the wild-type mES cells were killed. **(C)** The FACS analyses for the EGFP expression levels. In these FACS analyses, the EGFP expression level (FL1) under the selection with the minimal concentration (1×, green line), twice higher than the minimal concentration (2×, red line) and three times higher than the minimal concentration (3×, blue line) were analyzed by flow cytometry using FACSCalibur (BD). Blue filled peaks indicate the signals from wild-type mES cells. **(D)** Quantification of the relative cell numbers expressing EGFP. The proportions within M1 indicated in C are shown.

To evaluate the kinetics of the bi-cistronic expression vectors carrying various drug resistance genes in mES cells, we constructed a bi-cistronic expression vector system using *enhanced green fluorescent protein* (*Egfp*) as an indicator of the expression level and drug resistance genes under the control of the *IRES* from *encephalomyocarditis virus* (EMCV) driven by the *CAG* expression unit [10] (Figure 1A). To apply comparable selection pressures in different drug-selection systems, we first determined the minimal doses of each drugs to kill mES cells by serial titrations (Figure 1B). We determined the minimal doses as killing more than 93% of mES cells at low cell density (1×10^3^ cells per 90 mm dish) within 6 days, indicated with asterisks (Figure 1B). Each vector, that is, *pCAG-Egfp-IRES-neo*, -*hph*, -*zeo*, -*hisD*, -*bsd*, and -*pac*, was transfected independently into mES cells via random integration into the genome by electroporation, and the transfected cells were seeded at high density (1×10^6^ cells per 90 mm dish) and cultured in the presence of each drug at the concentrations of the minimal doses, twice higher or three times higher, from the next day. After the selection for 7 days, cells were harvested and seeded on new plates followed by the culture with the same concentrations of the drugs. Then the expression levels of the transgenes in the proliferated cells were estimated by the intensities of the fluorescence measured by flow-cytometry (Figure 1C) and the Egfp-positive proportions were scored (Figure 1D). Selection by *neo* showed broad and lower levels of fluorescence even at the highest dose (3 times higher than the minimal dose), in which less than 50% of the selected cells were diagnosed as Egfp-positive and strong positive cells expressing Egfp more than the relative intensity value of 10^3^ were merely observed. This situation may refer to the original distribution of the Egfp expression under the *CAG* unit at different integration sites in the genome. Similar tendency was observed for the selection with *hph*, *hisD* and *bsd* although the *hisD* selection gave higher levels of Egfp expressions and the *bsd* selection conferred higher proportion of the Egfp-positive cells than the *neo* selection. In contrast, selection with *pac* and *zeo* gave high proportion of Egfp-positive cells with high levels of fluorescent signals, indicating the enrichment of the transfectants expressing high levels of the transgene. This is not due to the application of the high doses of drugs because once the selection systems started to work at the minimal dose at high density culture condition, which was twice of the minimal doses we determined in the pilot experiments at low density culture condition, both *pac* and *zeo* selection systems gave the sharp enrichment of high expressants. Therefore, we suppose that this observation reflects the different threshold expression levels of the drug resistant genes to confer the drug-resistant phenotypes in mES cells. *Pac* and *zeo* require higher levels of expression to support the proliferation in the presence of the lethal amount of drugs than others, which allowed us to obtain the transfectants with homogenously high levels of transgene expression. These evidences accorded to our experiences that *zeo* and *pac* efficiently worked to select mES cell lines expressing fluorescent markers ubiquitously and strongly in chimeric embryos [11], and that *neo* gave higher numbers of LIF-independent colonies than *pac* when applied to select drive the expression of *Tbx3* transgene, of which the expression at high level is toxic in mES cells [12].

### Generation of new fusion genes of fluorescent markers and drug-resistant genes

**Figure 2:**
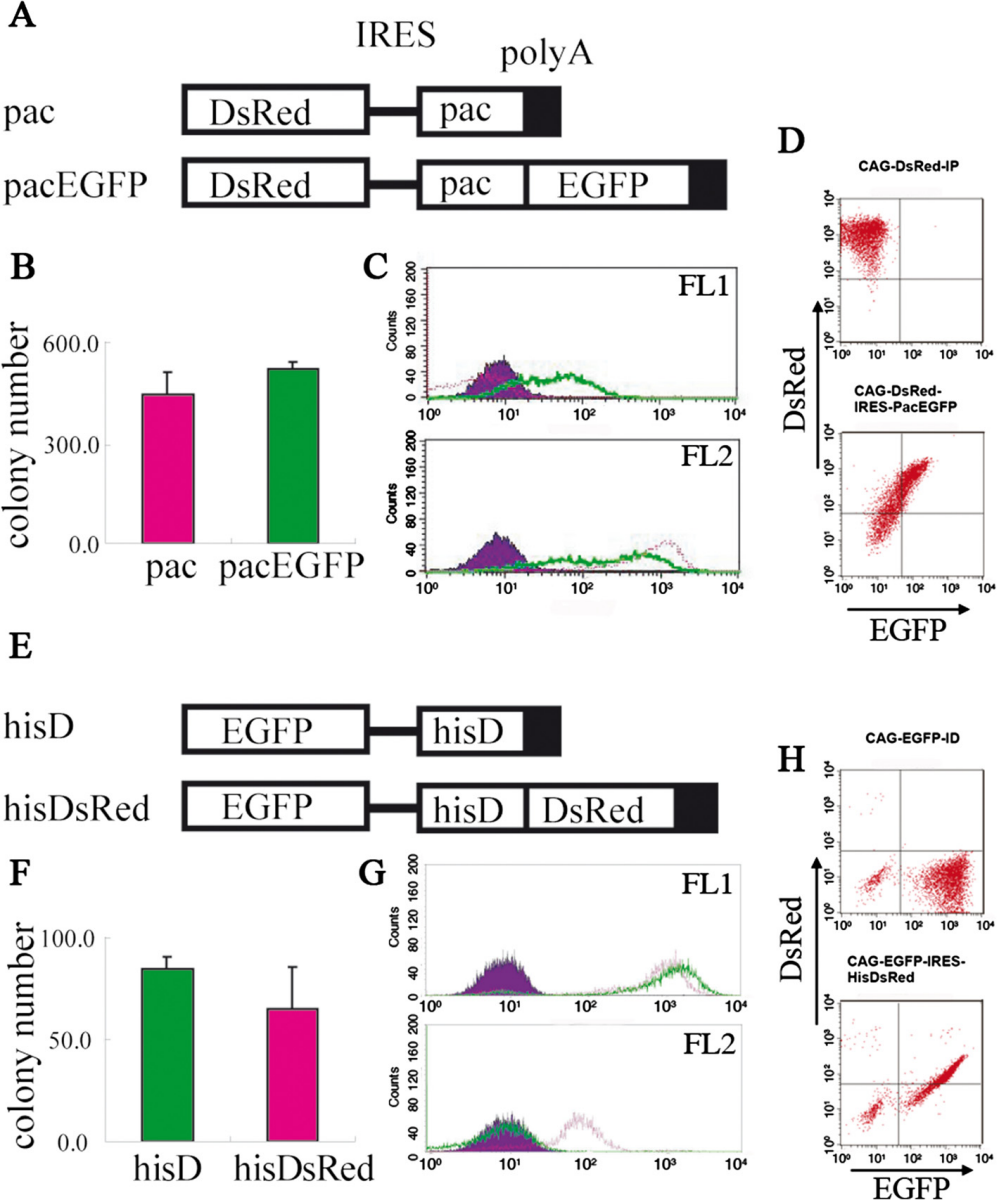
**Bi-functional chimeric proteins of drug resistance genes and fluorescent markers. (A, E)** Design of the vectors to test the functions of the fusion proteins driven by the CAG promoter. All constructs were electroporated into D3 mES cells, and the transfectants were selected with the standard concentrations of puromycin **(A)** or histidinol **(D)** as in Figure 1. **(B, F)** The numbers of the primary colonies from *pac* (red) and *pacEGFP* (green) after puromycin selection **(B)**, and *hisD* (green) and *hisDsRed* (red) after histidinol selection. Comparable numbers of drug resistant colonies were obtained in both cases by the fusion constructs. **(C)** FACS analyses of the transfectants with *pac* vectors. EGFP fluolescence could be detected in the transfectant containing *pacEGFP* (green line) but not *pac* (red line) (upper panel: FL1). Levels of DsRed fluorescence in ES cells with *pacEGFP* (green line) were comparable to that with *pac* (red line) (lower panel: FL2). **(D)** Correlation between the expression levels of DsRed and Egfp from *CAG-DsRed-IRES-pac-pA* (upper) and *CAG-DsRed-IRES-pacEGFP-pA* (lower). **(G)** FACS analyses of the transfectants with *hisD* vectors. Levels of EGFP fluorescence in mES cells with *hisDsRed* (red line) were comparable to that with *hisD* (green line) (upper panel: FL1). DsRed fluolescence could be detected in the transfectant containing *hisDsRed* (red line) but not *hisD* (green line) (lower panel: FL2). **(H)** Correlation between the expression levels of DsRed and Egfp from *CAG-EGFP-IRES-hisD-pA* (upper) and *CAG-EGFP-IRES-hisDsRed-pA* (lower).

If the modulation of the transgene expression levels by different drug concentration with the bi-cistronic expression vector enables us the precise control of transgene expression, the monitoring of the expression levels of the drug resistance genes in living cells will be ideal. The functional fusion genes of *Egfp* and *neo*, and *Egfp* and *hph* have already been reported [13]. Here we made two novel chimeric selection markers composed of drug resistance genes and fluorescent markers and tested their functions. Using the same system as described above, we confirmed that both *hisDsRed* (*hisD* + *DsRedT4*) and *pacEgfp* (*pac* + *Egfp*) are bi-functional as drug resistance markers comparable to the wild-type genes and as fluorescent markers (Figure 2A, E): The fusion genes worked as drug-resistant genes as efficiently as the wild-type genes since they gave comparable numbers of the drug-resistant colonies (Figure 2B, F); They also worked as the fluorescent markers that were expressed at the levels correlating to the transgenes placed at the upstream (Figure 2C, D, G, H). Therefore, these fusion genes will be useful for monitoring the gene expression levels in the stable transfectants.

### Application of the drug-resistant systems to gene targeting

**Figure 3:**
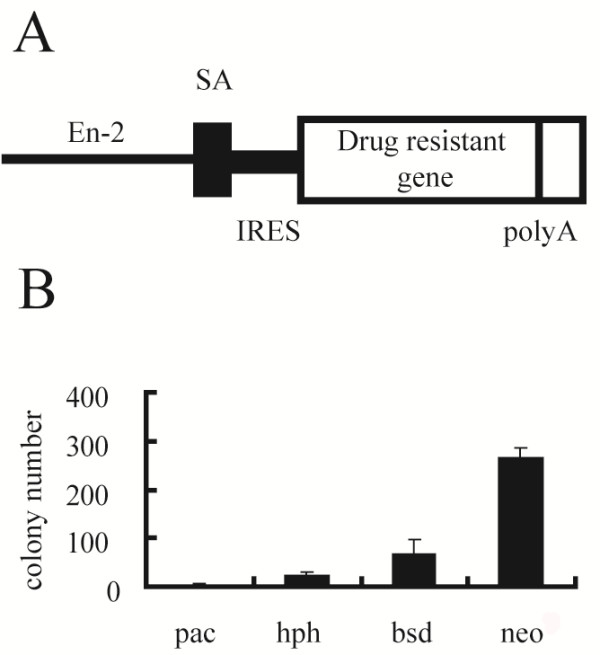
**Comparison of the efficiency of gene trap with different drug selection systems. (A)** Design of the gene trap vectors containing the different drug resistance genes. The drug resistance genes were fused to EMCV-IRES and placed downstream of the splice acceptor (SA) of *Engrailed* (*En*)-*2* gene [15]. **(B)** The numbers of drug-resistant colonies in gene-trap screening were counted in each drug selection. Columns and bars represent average colony numbers and standard error means (s.e.m.) among the triplicate per 2 × 10^7^ mES cells transfected with 100 μg of each promoter trap vector, respectively.

The *IRES*-mediated drug resistance gene cassettes are also useful for the gene targeting since this strategy is able to enrich the homologous recombinants [14]. When the promoter-less knockout vectors carrying the *IRES*-mediated drug resistance gene cassettes are integrated into the genome via random insertion, only the clones in which the *IRES*-mediated drug resistance gene cassettes are accidentally inserted into the genes and driven by the upstream promoter will survive, whereas all homologous recombinants are alive if the transcription levels of the *IRES*-mediated drug resistance gene cassettes reach the threshold levels to confer the drug resistance. To evaluate the expression levels achieved by these selection systems in comparison to the endogenous genes, we next tested their functions by the gene-trap method [15]. We placed the drug resistance genes fused to *IRES* (Figure 3A) downstream of the splice acceptor and introduced them into the host cells. With the *pac* selection system, we obtained very few drug-resistant colonies—less than 10% of the number obtained with the *neo* selection system (Figure 3B)—indicating that the threshold expression level of the pac selection system could be achieved only with strong endogenous promoters. Previous applications of the *neo* selection system to the gene-trap strategy in mES cells revealed that ~15,000 of ~20,000 genes expressed in mES cells could be trapped [16], indicating that this selection system can work under the control of endogenous promoters with weak transcriptional activities. We succeeded to obtain homologous recombinants for *Nrp1, Nrp2, Sema4d, Cry1, Cry2* and *Rad18* using the promoter-less targeting vector with *neo* at high efficiency (>10% of G418 resistant clones), confirming the high sensitivity of the *neo* selection system because the expression levels of these neuronal and housekeeping genes are moderate or low in ES cells (Table 1). In contrast, the promoter-less targeting vector with *pac* looks only applicable for the genes expressed at high levels, such as *Zfp42* and *Oct3/4*, although the knockout efficiency is high (>50% of puromycin resistant clones). In the case of *Estrogen-related receptor β* (*Esrrb)*, we got the homologous recombinants using the promoter-less targeting vector with *neo* but not with *pac*, indicating the difference of their threshold levels as well as the importance of their choice ( [17] and Sugimoto, Adachi and Niwa, unpublished). Other markers are also applicable for the promoter-less targeting vectors like in the cases of *Zfp42*, *Oct3/4* and *Sox2*, which are all expressed at high levels (Table 1).

**Table 1:** The expression levels of the genes targeted by the promoter-less vectors in ES cells

***Symbol***	**Average**	**Applied selection**	**Ref**
	log intensity*	**markers**	
***Zfp42***	**4.8854**	***pac, hph, bsd***	**Toyooka *et al.*, 2008 [**[18]**]**
			**Masui *et al.*, 2008 [**[11]**]**
***Socs3***	**4.4746**	***pac***	**(this paper)**
***Pou5f1***	**4.4563**	***pac, neo, zeo***	**Nichols *et al.*, 1998 [**[19]**]**
		***hph, bsd***	**Toyooka *et al.*, 2008 [**[18]**]**
***Klf4***	**4.2232**	***pac***	**(unpublished)**
***Sox2***	**4.2167**	***hph, hisD***	**Masui *et al.*, 2007 [**[20]**]**
***Sema4d***	**3.8042**	***neo***	**Taniguchi *et al.*, 2009 [**[21]**]**
***Nrp2***	**3.6699**	***neo***	**Takashima *et al.*, 2002 [**[22]**]**
***Cry1***	**3.5175**	***neo***	**Vitaterna *et al.*, 1999 [**[23]**]**
***Rad18***	**3.4387**	***neo***	**Tateishi *et al.*, 2003 [**[24]**]**
***Cry2***	**3.2127**	***neo***	**Vitaterna *et al.*, 1999 [**[23]**]**
***Esrrb***	**2.0747**	***neo***	**Martello *et al, *2012 [**[17]**]**
***Nrp1***	**1.6905**	***neo***	**Takashima *et al.*, 2002 [**[22]**]**

*Microarray data of 50 ES cell lines (Nishiyama et al., [25]). *Hprt*: 4.9305, *Gapdh*: 5.2380, *Actb*: 5.2452.

### Efficient selection of the gene conversion event by pac

**Figure 4:**
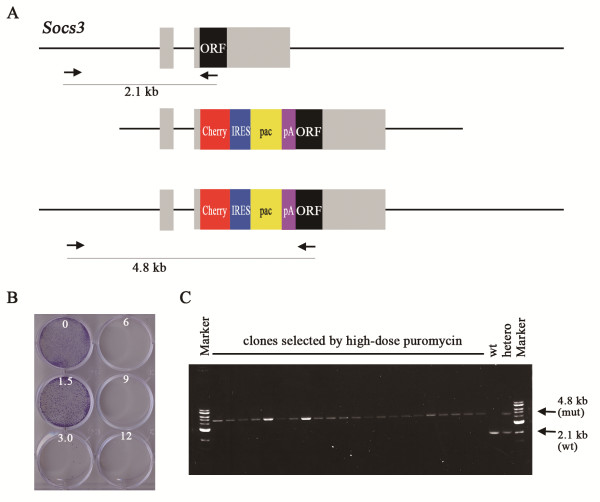
**Generation of mES cells homozygous for the Socs3 knock-out allele by high-dose puromycin selection. (A)** Strategy for the generation of *Socs3*-KO ES cells. The schematic maps of the *Socs3* allele (top), the KO vector carrying the *Cherry-IRES-pac-pA* cassette (middle), and the KO allele generated by homologous recombination (bottom). The PCR with the primers set at the 5′ external genome and the open reading frame (ORF) in the second exon provides the polymorphism between the wild-type and mutant alleles, 2.1 kb and 4.8 kb, respectively. **(B)** Sensitivity of the heterozygous clone to various concentration of puromycin. Most of the cells were killed by 3.0 μg/ml of puromycin for 5 days. **(C)** PCR analysis of the genotypes of the clones selected by 6 μg/ml of puromycin from the heterozygotes. All clones possess the mutant allele only, indicating that they are homozygotes for the *Socs3*-KO allele.

Sensitive dosage effect of the puromycin selection system could be advantageous for the tight selection of the expression level of *pac*. To select the homozygous mutant mES cells spontaneously appearing in the heterozygous pools via gene conversion, the efficient selection of the cells expressing two copies of the selection marker genes from those expressing one copy is required. The *neo* system was reported to be applicable for such selection, but the efficiency is not so high in general as originally reported [26]. We previously applied the puromycin selection system in the same way to get the homozygous mES cells for the *Zfp42* knockout allele, which resulted in the 100% efficiency of the selection (4 in 4 clones analyzed) [11]. Here we tested the general applicability of this strategy in heterozygous mES cells carrying the promoter-less *pac* cassette. In the case of the heterozygous mES cells for the *Socs3* knockout allele, the titration of puromycin concentration determined the maximal dose allowing the survival of the majority of the heterozygotes at 4 μg/ml, and the high-dose (6 μg/ml) puromycin selection resulted in the 100% efficiency of the homozygous mutant selection (46 in 46 clones analyzed; Figure 4). Similarly, in the case of the heterozygous mES cells for the floxed *Klf4* allele, the high-dose (6 μg/ml) puromycin selection resulted in 60% efficiency of the homozygous mutant selection (12 in 20 clones analyzed; data not shown). These data indicated that the tight dosage-dependency of the puromycin selection system is suitable for this strategy.

## Discussion

The drug resistance genes described here can be used simultaneously for multiple transgene expression and gene targeting in mES cells. There is no interference between any of these positive selection markers because we succeeded to obtain mES cells carrying multiple drug-resistance genes in various combinations [20,27]. Here we described the different kinetics of the drug selection systems *pac, zeo, hph, hisD, bsd* and *neo*. *Pac* and *zeo* require high levels of expression to confer the proper drug resistance to mES cells, whereas *neo* establishes the resistance to G418 with minimal expression level in mES cells.

In the conventional transgene expression strategy, the drug resistant genes were driven under the control of strong viral enhancer/promoters derived from simian virus 40 and human cytomegalovirus. However, separation of the expression units of the transgene and the drug-resistant genes failed to ensure the transgene expression in the drug-resistant cells and it was required to screen a large number of drug-resistant clones to obtain the stable transfectants with ideal expression levels. The *IRES*-mediated bi-cistronic expression vector directs the expression of the transgene and the drug-resistant gene from the same promoter, in which the drug selection always confirms the expression of the transgene. If we apply the rule we identified in this report, it is possible to design an expression vector with an ideal expression level of the transgene by a proper choice of the drug selection system. To obtain the homogenous expression of the fluorescent markers in mES cells, *pac* and *zeo* are recommended as we succeeded previously [11,20]. However, when a moderate or low level of expression is ideal like in the cases of tetracycline-dependent transcriptional activator tTA/rtTA, of which the expression at a high level is toxic for mES cells (Niwa, unpublished), and Tbx3 [12], *neo* is the first choice.

We recently reported the function of *Esrrb* as a target of glycogen synthase kinase-3 (Gsk3)-Tcf3 pathway [17]. In this report we first applied the bi-cistronic expression vector with *CAG* and *IRES-hph* to direct the expression of *Esrrb* in mES cells, resulting in 6–8 fold higher expression of *Esrrb* transgene than the endogenous levels. Since this situation creates the possibility of neomorphic effects, we switched to the expression vector with *IRES-neo* and succeeded to confirm that the constitutive expression of the exogenous *Esrrb* at endogenous levels is sufficient to sustain self-renewal of mES cells in Gsk3 inhibitor-independent manner. This is a good example demonstrating the importance of the choice of a proper drug selection system to obtain appropriate levels of transgene expression. Well-designed strategy for transgene expression will provide clear results in cell biological analyses.

## Conclusions

The expression levels of the transgenes using the bi-cistronic expression vectors depend on the drug selection systems. Appropriate choices of the systems will give clean results. This is also applicable to the gene targeting with bi-cistronic durg-resistant genes. The principle shown here in mES cells should be applicable to mouse induced pluripotent stem (iPS) cells directly and most likely to human ES cells after modification.

## Methods

### Plasmid constructions

Initial Methionine of all drug resistance genes in Egfp expression vectors were fused in frame to ATG sequence of the *NcoI* site in 3′ terminus of EMCV-*IRES* sequence derived from *pCITE-1* (Novagen). *pCAG-IP* and *pCAG-IZ* plasmid was constructed for puromycin and zeocin selection as described [9,28]. *pCAG-IB* was constructed by replacing the *NcoI-XbaI* fragment in *pCAG-IZ* with the *BsaI-SpeI* fragment containing the *bsd* gene derived from *pUC-SV-BSD* (Funakoshi). *hisD* ORF was amplified from *pAGHisD* plasmid (a gift from S. Takeda, Kyoto university) by PCR method with sense primer fused to *BspHI* recognition site and antisense primer fused to *XbaI* site and exchanged *NcoI* and *XbaI* fragment of *pGTIRESβgeopA*[14], resulting *pGTIRESHisDpA*. *pCAG-ID* was made by replacement of *KpnI-BamHI* fragment between the *pGTIRESHisD* and *pCAG-IP*. *PvuI-MscI* fragment of *pBR322* was ligated to blunted *BamHI* and *PvuI* digested *pCAG-IP*, resulting *pBRCAG-IP*. *pGTIRESHygropA* was made by exchanging *BspHI-BglII hygro-PGKpA* fragment from *pSP72-tkphygropA* with *NcoI-BamHI* fragment of *pGTIRESβgeopA* in which *BamHI* and *BstXI* sites within *PGKpA* are disrupted. *pCAG-IH* was constructed by exchanging *BamHI-KpnI* fragment between *pBRCAG-IP* and *pGTIRESHygropA*. The *puromycin resistance gene* of *pBRCAG-IP* was also exchanged with PCR fragment amplified from *pMC1-neo-pA* (Stratagene) with *BspHI* site attached sense primer and both *BamHI* site and SV40 polyA attached antisense primer, resulting *pCAG-IN*. *pPyCAGIHisDsRedT4* was made by fusing of *DsRedT4*[29] open reading frame (ORF) to C-terminus of *hisD* gene linked with 5′-gagcaagcaagatcgaccaccatg-3′ sequence. The *Egfp* fragment with *XhoI* and *NotI* site obtained from *pEGFP-N1* (Clonetech) by PCR was inserted between *XhoI* and *NotI* site upstream of *IRES* in each expression vector backbone, *pCAG-IP, -IZ, -ID, -IH, -IB*, and –*IN*, examined for puromycin, zeocin, histidinol, Hygromycin B, blasticisin S, and G418 selection, respectively. *pCAG-IpacEGFP* was constructed by replacement *pac* fragment of *pCAG-IP* with *pacEGFP* fusion fragment ligated between *SalI/NotI* digested *Egfp* fragment from *pEGFP-N1* and *pac* fragment amplified by PCR (sense: 5′-CCTCATGACCGAGTACAAGCCCA-‘3 antisense: 5′-CGGATCCGGCACCGGGCTTGCGGGTCAT-3′) that was digested with *BspHI/BamHI*, linked between partially filled *BamHI* and *SalI* site. *hisDsRedT4* ORF was inserted between *XhoI* and *NotI* site of *pCAG-IP* and –*IpacEGFP*. Full sequence information’s of all expression vectors are available on our web site (http://www.cdb.riken.jp/pcs/protocol/vector/vector_top.html).

### Cell culture and electroporation

D3, E14tg2a and EB3 ES cells were maintained in the absence of feeder cells in Glasgow minimal essential medium (GMEM) supplemented with 10% fetal calf serum, 10^-4^ M 2-mercaptoethanol, and 1000 unit/ml of LIF at normal condition 37°C, 5% CO2 [30]. 2 × 10^7^ ES cells were electroporated with 30 μg of linearized plasmid DNA at 800 V and 3 μF in a 0.4 cm cuvette using a Gene-Pulser (Bio-Rad) and then cultured in the presence of the drugs for selection, Puromycin (Nacalai tesque) Zeocin (Invivogen), Hygromycin B (HygroGold, Invivogen), L-Histidinol (Sigma), Blasticidin S (Invivogen) and G418 (Nacalai tesque), at indicated concentrations. Colonies were identified by Leishman (SIGMA) staining, and counted.

### Flow cytometric analysis

Transfectants grown in the presence of each drug concentrations were harvested and 10.000 data points were collected for each sample in flow cytometry, using FACSCALIBUR (Becton Dickinson). Data were analysed using CellQuest Pro Software ver.5.2 (Becton Dickinson).

### Gene targeting of Socs3

Genomic DNA sequences were amplified using the primers 5′- ataaatCGatGGCGGCTCTAACTCTGACTCTACACTC-3′ and 5′- ttaagctTGGCGCACGGAGCCAGCGTGGATCTG-3′ (for the left arm of the KO construct); and 5′- CCGGGATcCGGTAGCGGCCGCTGTGCGGAG-3′ and 5′- CAGAGCTCgtcgaCTCCTGTCTGTACAGAAGGAAAGAGAGAG-3′ (for the right arm of the KO construct). Amplified PCR products were cloned into *pBR-blue* vecotor. The 1.0 kb *ClaI* (in primer)–*NotI* (in genomic DNA) fragment from the left arm and the 3.0 kb *NotI* (in genomic DNA)–*SacI* (in primer) fragment were cloned into *pBR-MC1DTApA*. The *NotI* site was used to clone the marker gene cassette *dCherry-IRES-pac-pA*. 1 × 10^7^ EB3 ES cells were electroporated with 100 μg of plasmid DNA linealized by *XhoI*. From the next day, these transfectants were selected with 1.5 μg/ml of puromycin for 8 days. 16 puromycin-resistant colonies were picked, expanded and analyzed their genotype by PCR using the primers 5′- CAGTCCTCCTAGTCGACATTCCTTCTC-3′ 5′- ttaagctTGGCGCACGGAGCCAGCGTGGATCTG-3′ with KOD-Fx (Toyobo) that amplify 2.1 kb fragment from the wild-type allele and 4.8 kb fragment from the targeted allele. One of three homologous recombinants (sKO2) was examined for their ability to survive higher concentration of puromycin, and 1 × 10^6^ sKO2 ES cells were selected with 6 μg/ml of puromycin for 4 days followed by the culture with 1.5 μg/ml of puromycin for 6 days. About 100 colonies were formed and 46 clones were picked, expanded and analyzed their genotype by PCR using the primers shown above.

## Competing interests

The authors declare they have no competing interests.

## Authors’ contributions

YN and HN conceived the project and designed the experiments along with all authors performed the experiments. YN and HN wrote the manuscript. All authors read and approved the final manuscript.
